# Spontaneous intracranial hypotension: Two cases including one treated with epidural blood patch

**DOI:** 10.4103/0972-2327.56318

**Published:** 2009

**Authors:** Pankaj Agarwal, Suresh Menon, Rajan Shah, B. S. Singhal

**Affiliations:** Departments of Neurology, Bombay Hospital Institute of Medical Sciences, Mumbai, India; 1 Departments of Neurosurgery, Bombay Hospital Institute of Medical Sciences, Mumbai, India

**Keywords:** Epidural blood patch, spontaneous intracranial hypotension

## Abstract

Spontaneous intracranial hypotension (SIH) is characterized by orthostatic headache (OH), low cerebrospinal fluid (CSF) pressure, and diffuse pachymeningeal gadolinium enhancement (DPME). We present here the case studies of two patients. One patient demonstrated a CSF leak in the mid-thoracic region, and recovered completely with conservative treatment. The other patient in whom leak could not be demonstrated, developed dementia, rapidly worsening encephalopathy, and became comatose, necessitating urgent epidural blood patch (EBP) with 25 cc of autologous blood, after which immediate and complete symptomatic relief was obtained. A second EBP was required a few days later and also provided complete and sustained clinical benefit, without subsequent recurrence. Both patients had OH and showed bilateral subdural fluid collections, DPME and “sagging” of brain on MRI. A high index of suspicion, recognizing the orthostatic nature of headache, and typical findings on contrast enhanced MRI should point to the diagnosis of SIH. EBP can be effective treatment in patients unresponsive to conservative measures.

## Introduction

Spontaneous intracranial hypotension (SIH) or syndrome of spontaneous cerebrospinal fluid (CSF) hypovolemia is characterized by orthostatic headache (OH), low CSF pressure, and DPME (diffuse pachymeningeal gadolinium enhancement) on magnetic resonance imaging (MRI), in the absence of head trauma or lumbar puncture.[1,2] OH, as defined by the International Headache Society, is a headache that occurs within 15 minutes of an upright position and is relieved within 30 minutes of recumbency.[3] Almost all cases of SIH are a result of spontaneous spinal CSF leakage from spinal meningeal diverticula or simple dural tears.[1,2] Epidural blood patch (EBP) is the mainstay of treatment when conservative measures fail. Although well described from other parts of the world, there exists a remarkable paucity of reports of SIH from India. We could identify only one case series of this condition from India[4] and no report of a patient treated with EBP. We report two cases of SIH, one of whom was treated successfully with EBP.

## Case Reports

### Case 1

**Figure 1:**
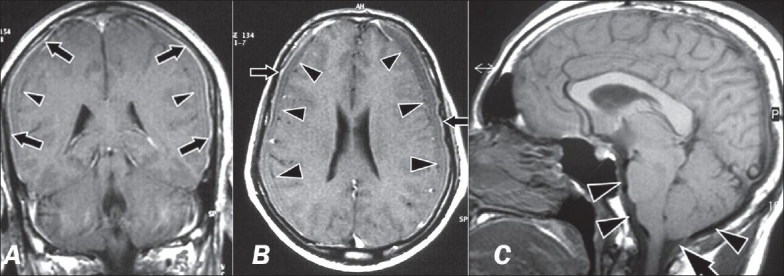
Composite figure showing classic MRI features of SIH. Gadolinium enhanced T1 weighted coronal (A) and axial (B) MRI brain showing diffuse pachymeningeal enhancement (arrows) and bilateral thin subdural hygromas (arrowheads) over cerebral convexities (Case 2) (C): Midsaggital T1W image shows downward descent (“sagging”) of brainstem and cerebellum, with flattening of the pons, narrowing of the prepontine cistern (arrowheads) and descent of cerebellar tonsils (arrow). (Case 1)

**Figure 2:**
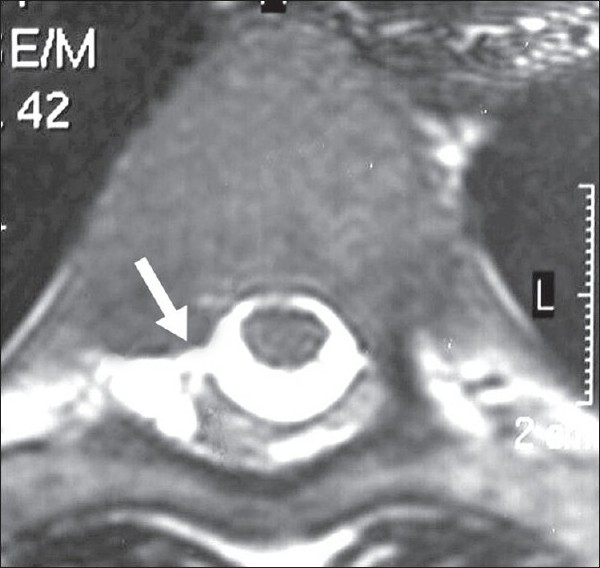
Axial T2 weighted MRI spine at T6 vertebral level showing a right paraspinal extra-arachnoid CSF collection with a communicating tract (arrow) to subarachnoid CSF (Case 1)

A 25-year-old executive presented with a three-year history of migraine. He had been experiencing a different type of a more severe, persistent, nuchal and occipital headache since the last three weeks. The pain was nonthrobbing and unassociated with nausea or vomiting. It had developed over a week and had slowly grown in intensity, and had begun to bother his work while he sat in his office chair. It would begin within 15 minutes of sitting or standing, and subside within 30 minutes after lying down. He would notice a peculiar feeling of “something moving inside his head” when he would sit up or stand. The past history was remarkable for a shoulder dislocation suffered three years ago. Examination showed subtle marfanoid features such as tall stature and long slender fingers. Neurological examination was normal. MRI brain showed bilateral subdural hygromas, “sagging” of the brainstem [Figure 1], DPME, and engorged venous sinuses. Routine MRI spine revealed a CSF leak at T6 vertebral level with a localized CSF collection in the posterior right epidural space [Figure 2]. The patient was treated with bed rest and liberal fluid intake for four weeks with complete symptomatic relief. He was able to resume his normal activities thereafter and had no recurrence of symptoms. One year later, follow-up MRI showed almost complete resolution of subdural collections, and significantly less brainstem “sagging”. MRI spine findings, however, remained unchanged.

### Case 2

A 50-year-old male presented with a new onset, diffuse bifrontal and occipital, orthostatic headache since two months. Headache would worsen markedly within a few minutes of assuming an upright position. He had become progressively dull and apathetic, and was incontinent for urine since the last few weeks. He had vomited twice in the last week. On examination, he was conscious, but withdrawn and inattentive. Fundi were normal, lower limb reflexes were exaggerated and plantars were extensor. Rest of the neurological examination was normal. MRI brain showed bilateral thin subdural fluid collections and DPME [Figures 1B and 1C]. Routine MRI spine did not reveal a CSF leak. He was treated with bed rest and hydration. A few days after admission, he became progressively more drowsy, and later unresponsive, with poorly reactive pupils, and a decerebrate response of the left upper limb to pain. As an emergency measure, 25 cc of autologous blood was injected into the L3-L4 epidural space, followed by placement in the Trendelenburg position. Within a few hours, he became fully conscious, alert and responsive. Four days later, he became drowsy again. A repeat EBP with 30 cc of blood was performed, with improvement in sensorium over the next few hours. There was no subsequent recurrence and he remained asymptomatic thereafter.

## Discussion

Both our patients had orthostatic headache (OH) as defined by the International Headache Society, without prior head trauma or lumbar puncture. In both cases, we observed subdural fluid collections and DPME on MRI, the two most common imaging features of SIH.[2] Downward descent (sagging/sinking) of the brain with effacement of subarachnoid cisterns and crowding of posterior fossa is a specific imaging feature of SIH,[2] and was also seen in both patients. The headache is a result of downward displacement of the brain due to loss of CSF buoyancy, causing traction on pain-sensitive structures, particularly the dura. Though classically ‘orthostatic’, headache in SIH may be of a variety of patterns: non-orthostatic, chronic daily, exertional, acute “thunderclap”, paradoxical (increased on recumbency) or even absent.

In the first patient (Case 1), routine MRI spine revealed a CSF leak at the level of the sixth thoracic vertebra. The thoracic spine is the most common site of a CSF leak in SIH.[1,2] However, in most cases, MRI may show only extra-arachnoid fluid collections over several levels, or CSF extravasation into paraspinal soft tissues over fewer levels. Demonstration of the exact site of the leak, such as was seen in our case, is uncommon. A year later this MRI spine finding remained unchanged despite disappearance of clinical symptoms and improvement of brain imaging findings. It is conceivable that a degree of functional “closure” of the dural rent had occurred due to alteration of CSF flow dynamics over time, although its anatomic correlate had curiously remained unchanged. It is also intriguing that this patient with the larger, easily demonstrable, persistent CSF leak improved with bed rest, while the other patient (Case 2), in whom leak could not be demonstrated, worsened rapidly and needed treatment with EBP. More sensitive tests such as spinal CT myelography and radioisotope cisternography would have been useful in demonstrating the location of CSF leak[1,2] in Case 2. However, we were unable to perform these tests because of rapid clinical worsening a few days after admission, necessitating urgent therapeutic intervention.

Other common symptoms in SIH include neck/back pain and nausea (noted by our patients), altered sound perception, dizziness, diplopia and other visual, facial sensory, and radicular limb symptoms. Although rare, a reversible frontotemporal pattern of dementia has been described in SIH.[5] Reversible encephalopathy, stupor, and coma due to diencephalic compression have also been reported in several patients.[6] Case 2 in our study had developed a similar predominantly frontal lobar affection with apathy and abulia, and went on to become comatose with signs of brainstem herniation. All these features resolved promptly and completely after EBP.

The first patient (Case 1) had a history of shoulder dislocation a few years ago, and in him we noted marfanoid features. Approximately one-fifth of patients with SIH have subtle skeletal manifestations of Marfan syndrome, such as tall stature, arachnodactyly, high arched palate, and joint hypermobility, but none of the other stigmata of the syndrome.[7] These patients do not harbor fibrillin gene mutations, but a defect of microfibrils, important components of extracellular matrix associated with fibrillin, has been demonstrated.[7] Other connective tissue disorders may also predispose patients with SIH to dural weakness, and hence to a spinal CSF leak.[2]

The underlying mechanism of the syndrome of SIH is probably neither CSF hypotension nor CSF hypovolemia per se, but rather an altered distribution of craniospinal elasticity due to spinal CSF loss, and “spontaneous spinal CSF leak” seems the preferred descriptive term.[8] We did not perform a CSF study on our patients as we felt that additional violation of the dura could worsen the underlying CSF leak.

All cases are usually treated conservatively with bed rest (which is also what the patient prefers anyway) and adequate hydration.[1,2] The leak may stop spontaneously in some patients, who recover regardless of treatment. Caffeine, theophylline and corticosteroids are also tried but no approach is of proven efficacy. Given time, these conservative measures are probably effective in many patients. EBP is the definitive treatment in those who fail to respond to conservative measures.[1,2] 20 cc of autologous blood is injected into the lumbar epidural space, after which the patient is placed in the Trendelenburg position for approximately two hours.[9] This allows the blood to ascend over several segments to seal the leak. The effect of EBP is twofold: an early effect related to volume replacement resulting from dural tamponade, and a latent effect that results from sealing of the leak.[9] The patient we treated with EBP probably benefited from both these mechanisms, as he had both immediate and long-lasting symptomatic relief. In spontaneous CSF leaks the success rate with each EBP is approximately 30%, while in CSF leaks following lumbar puncture, where the exact site is known and the anatomical defect is relatively simple, each EBP has a 90% chance of being effective.[9] A larger volume of blood (20–100 ml) may be used in cases that fail to respond to the initial patch, but this may cause back pain and radiculopathy. If the exact site of leak is known, a directed EBP or percutaneous placement of fibrin sealant may be of help.[2] Surgical repair of the dural defect may be considered in patients who fail two to three EBPs, and works best in cases where a structural defect or a focal CSF leak is identified. Suturing a leaking meningeal diverticulum or a dural rent, or closing a dural hole by placement of a muscle pledget, can be performed.[2] A transient rebound intracranial hypertension may be seen in some patients.[1] Recurrence of headache is seen in 10%.[2] The prognosis in most cases is good, as we noted in both our patients.

In summary, we describe clinical features, MRI findings, treatment and outcome in two cases of SIH. Reversible dementia, encephalopathy, and coma may rarely be encountered in SIH. A high index of suspicion, recognizing the orthostatic nature of headache, and typical findings on contrast enhanced MRI, such as bilateral sudural effusions and diffuse pachymeningeal enhancement, should lead one to the diagnosis. In patients not responding to conservative measures, EBP may provide effective treatment.
